# Electrospray deposition of physical unclonable functions for drug anti-counterfeiting

**DOI:** 10.1038/s41598-024-63834-x

**Published:** 2024-06-10

**Authors:** Bryce J. Kingsley, J. David Schaffer, Paul R. Chiarot

**Affiliations:** 1 Department of Mechanical Engineering, State University of New York at Binghamton, Binghamton, NY 13902 USA; 2 Institute for Justice and Well-Being, State University of New York at Binghamton, Binghamton, NY 13902 USA

**Keywords:** Mechanical engineering, Health care, Public health, Mathematics and computing

## Abstract

In recent years, pharmaceutical counterfeiting has become an increasingly dangerous situation. A patient who unknowingly consumes a counterfeit drug is at a serious health risk. To address this problem, a low-cost and robust approach for authentication that can be administered at the point-of-care is required. Our proposed solution uses Optical Physical Unclonable Functions (PUFs); patterns formed by a stochastic process that can be used for authentication. We create edible PUFs (ePUFs) using electrospray deposition, which utilizes strong electric fields to atomize a liquid suspension into a plume of micro-scale droplets that are delivered to the target. The ePUFs are electrospray-deposited from an edible ink directly onto the surface of the drug tablets. The process parameters (flow rate, translation speed, and suspension concentration) govern the characteristics of the ePUF to provide highly stochastic patterns. To evaluate our approach, 200 ePUFs were deposited onto tablets at various conditions, followed by imaging and storage of the patterns in a database. For ePUF authentication, a machine vision approach was created using the open source SIFT pattern matching algorithm. Using optimized pattern-matching constraints, our algorithm was shown to be 100% successful in authenticating the cellphone images of the ePUFs to the database. Additionally, the algorithm was found to be robust against changes in illumination and orientation of the cellphone images.

## Introduction

Drug counterfeiting has become increasingly problematic and presents a significant threat to the health and well-being of the general public. According to the Drug Enforcement Administration (DEA) of the United States, 9.6 million counterfeit pills were seized over a one-year period between April 2020 to April 2021; more than the two previous years combined. For an unknowing patient, consuming a counterfeit drug can lead to serious, or even deadly consequences if the drug is incorrect and/or contaminated with a harmful substance. This issue is ever more prevalent now with the abundance of online pharmacies which may not be licensed and could be selling products from non-reputable sources. Thus, there is increased demand for low-cost and robust identification technologies to be able to differentiate genuine drugs from those that are counterfeit.

There are various methods for identifying counterfeit drugs. The most-robust approaches use analytical chemistry techniques such as chromatography, electrophoresis, or spectroscopy (Raman, NMR, FTIR) to extract the chemical signature of the ingredients within the drug^1^. These techniques can be highly accurate, as small changes in the chemical makeup will show up in the chemical signature and the drug can be flagged. However, they often require specialized tooling which can make it slow and expensive especially if large batches of drugs need to be tested. Some manufacturers have started to include package-level (i.e., pill bottle) identification using barcodes or radio frequency identification (RFID) chips^2–4^, which identifies the package and assumes the pills within the package are all genuine if the package is tested to be authentic. This type of authentication fundamentally depends on the original packaging and no longer works if the contents are repackaged into smaller unit-of-use containers by distributors, which is a viable entry point where counterfeit drugs may find their way into the consumer market. Thus, the optimal drug authentication technology works using a *per-dose* (on-dose) identification scheme such that each pill is uniquely marked by the manufacturer and can be singly verified as genuine, which does not require the original packaging. However, per-dose drug authentication exposes additional challenges. Most importantly, the identification on each dose must be ingestible or easily removable, so that the drug can be safely consumed by the patient. Additionally, the identification should be robust and long-lasting, as to not diminish or deteriorate over extended periods of time, which could impact authentication validity.

In recent years, much research has been focused on developing and advancing digital authentication technologies to help combat counterfeiting of drugs and other consumer products. Some methods utilize an embedded tagging scheme, in which the identification tag is located inside the bulk of the material. Many of these embedded approaches use a chemical tag, such as a sequence-coded polymer^5,6^ which require specialized tooling to be able to read the tag for authentication. Han et al.^7^ proposed an alternative embedded technique which utilizes lithographically-encoded QR codes on polymer tags that can be inserted inside drug casings. Using the QR coded tag instead of a chemical tag removes some of the equipment constraints to be able to read it, however the tag still needs to be removed from the drug to be able to image it, which is not ideal. Thus, surfaced-based identification tags have become more popular since they are readily exposed and easier to capture for authentication. Some examples of surface-based tagging methods include: inkjet-printed QR codes^8,9^, wrinkle-based polymer micro-fingerprints^10^, and digitally-encoded polymer microfibers^11^ and metal nanomaterials^12,13^.

With any identification tagging method, the security and clone-ability is of great concern. Methods that use printed codes (i.e., QR codes) can be susceptible to security issues, as the codes can be cloned by bad actors with adequate printing equipment. One way to ensure strong security is to utilize Physical Unclonable Functions (PUFs). First introduced by the work of Pappu et al.^14^, PUFs have been used extensively over the past 20 years for authentication of silicon-based devices in electronics ^15–17^, and PUF identification is now branching out into other scientific fields, such as biology^18,19^. Recent work by Esidir et al.^20^ shows how electrospray deposition can be used as a tool to deploy PUF patterns as a means for anti-counterfeiting. In electrospray deposition a strong electric field is used to atomize a liquid precursor solution into a cloud of microdroplets. Electrospray deposition has been widely used for micro- and nano-manufacturing of structures and thin films^21–31^, and offers a promising PUF manufacturing technique given its stochastic nature and large parameter space for PUF variability.

**Figure 1:**
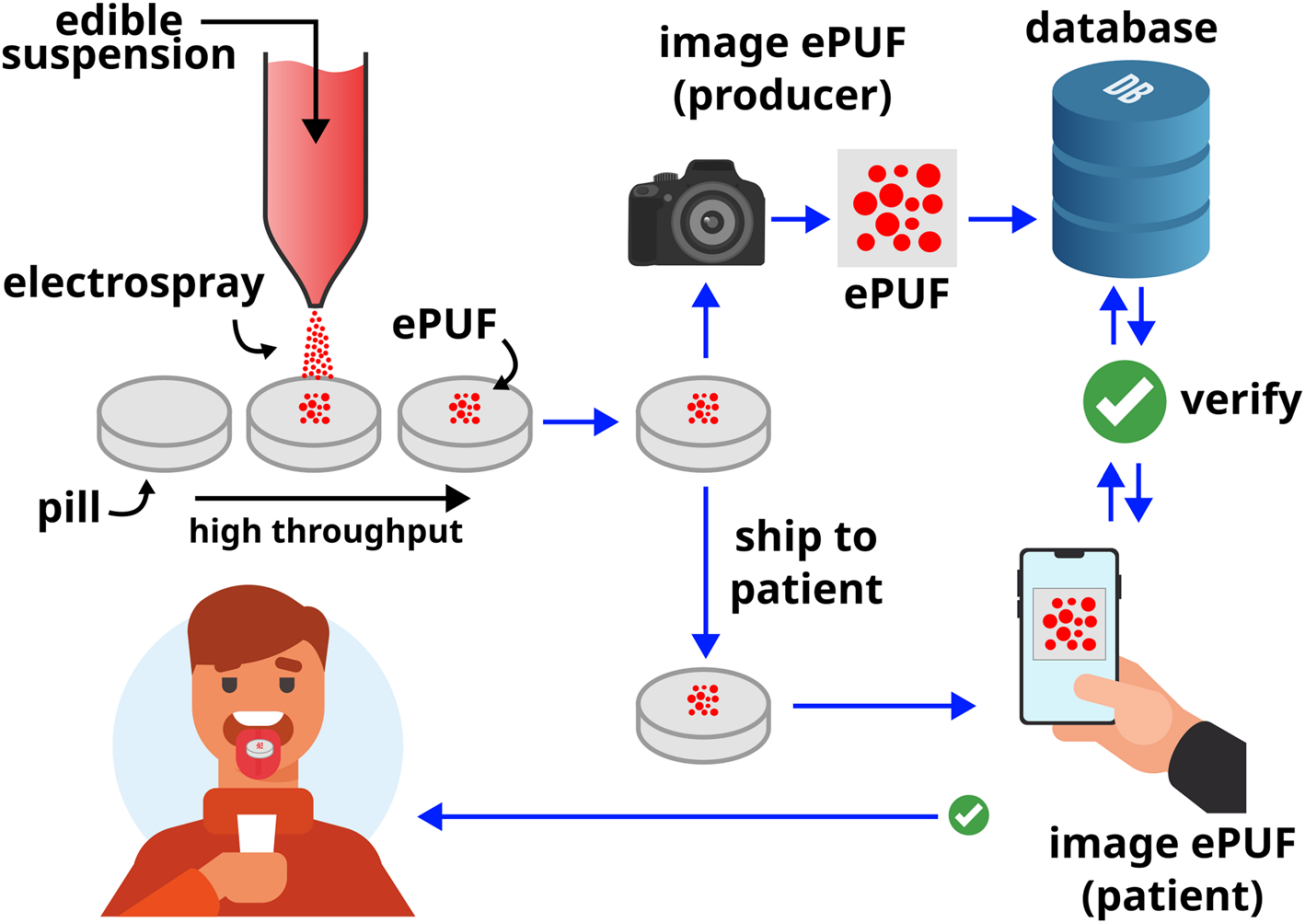
Schematic of electrospray-deposited edible-PUF (ePUF) onto drug tablets for per-dose authentication. ePUFs are deposited onto the pills using electrospray. Standardized images of the ePUFs are captured and stored in a database before the pill is shipped to the patient. The patient captures an image with their cellphone camera which is checked against the database to authenticate the pill.

In this work, we electrospray-deposit edible-PUFs (denoted as *ePUF*) onto drug tablets for identification and anti-counterfeiting protection. Here, we deploy optical PUFs ^32^ that are visible to the naked eye and can be imaged with a standard cellphone camera. This is in contrast to micro- or nano-scale PUFs that require electron/optical microscopy or other specialized tools for imaging and authentication ^13,17,20,33^. Optical ePUFs are appealing for consumers that are unlikely to have access to the specialized tools that would be required for micro-/nano- PUFs. Figure 1 shows an overview of the ePUF deposition and authentication process.

To ensure that the PUFs were safe for ingestion, an edible ink was formulated using a naturally-derived red food coloring dye and was used to create the ePUFs on the drug tablets. Standardized images of the ePUF-labeled drug tablets were taken and stored in a database for authentication. To authenticate the labeled drugs against the database, a machine-vision pattern matching algorithm was developed which matches a cellphone-captured image of an ePUF-labeled drug to its corresponding database entry, as long as it is valid. The pattern matching algorithm can be tuned to achieve very high matching accuracy, and is robust against variations in cellphone image quality, illumination, and orientation. Our ePUFs are electrospray-deposited directly onto the surface of the drug, in contrast to other approaches where the ePUF is fabricated on an adhesive carrier substrate that is then attached to the drug^34^. The latter method is unique in that the ePUFs can be fabricated independent of the drug, and then attached before the drugs are distributed. However, it exposes a potential security risk if the adhesive ePUF can be removed and re-attached to a counterfeit drug. By depositing the ePUF directly onto the drug, it cannot be removed or transferred, making it more secure. To employ this process in the broad consumer market, we envision that the drug manufacturer would fabricate (i.e., electrospray deposit) and image the ePUFs before the drugs are distributed, and the ePUF images would be stored in a secure database (proprietary or shared among manufacturers). The patient (i.e., drug consumer) could then use a cellphone application to capture an image of the drug and authenticate with the database to identify that the drug is safe for consumption.

## Materials and methods

### Materials for ePUF fabrication

Edible inks for ePUF deposition were prepared from a mixture of deionized (DI) water and Carmine, a natural red dye derived from the cochineal insect commonly labeled as cochineal extract or natural red 4. The Carmine dye was purchased from VWR in a powdered form. To prepare the inks for electrospray, Carmine was dissolved in DI water at 1 or 2 wt% solids. The Carmine and DI water solutions were suspended in an ultrasonic bath for 5 min to promote dissolution and homogeneity. Due to the low mass loadings (2 wt% max) of the inks, their physical properties (viscosity, surface tension, etc.) were comparable to that of DI water. Carmine was selected as the dying agent due to its solubility in water and deep red color. However, other dying agents can be substituted for Carmine with no change to the rest of the process.

Circular antacid tablets (16 mm diameter) were used as a model pill for ePUF deposition and analysis. These tablets were selected due to their low cost and moderate size, making them ideal for evaluating this technology. However, we note that this technology can also be applied to pill tablets of different sizes and shapes with only minor modifications to the overall process. The size of the deposited ePUFs is governed by the opening in the stencil used in electrospray deposition (discussed in the next section). Thus, smaller ePUFs can be fabricated by reducing the size of the stencil opening in order to target smaller pills.

### ePUF fabrication using electrospray deposition

**Figure 2:**
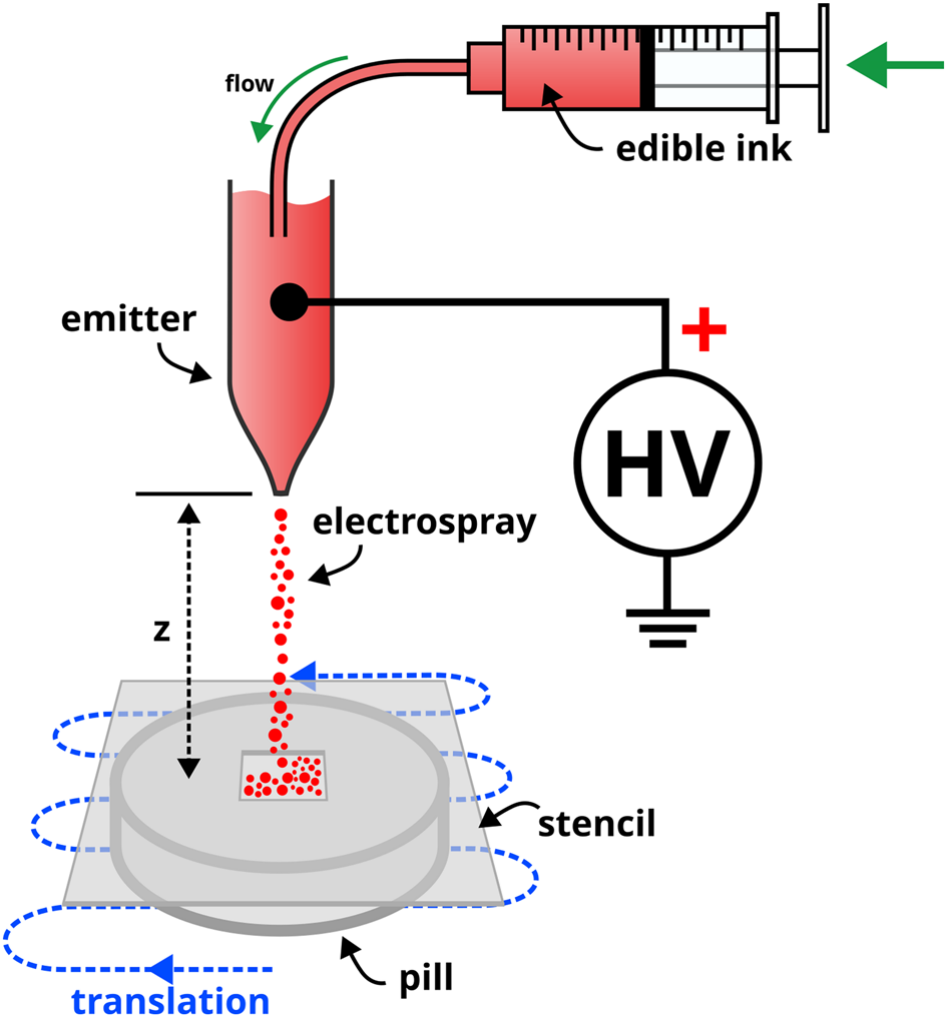
Schematic of ePUF deposition onto a pill. High voltage is applied to the edible ink solution to generate the electrospray. A plastic stencil is placed on top of the target pill, which is translated in a zig-zag pattern to deposit the ePUF on its surface through the opening in the stencil.

A schematic of ePUF deposition is presented in Fig. 2. The edible precursor ink is contained within a syringe attached to a syringe pump (not shown) to supply a continuous flow of solution. Flow rates for deposition ranged from 5 to 22 µL/min. The flowing liquid is supplied to an emitter (made in-house from borosilicate glass; 150 µm orifice ID) that is energized with a high voltage power source (Spellman) to generate the electrospray. Applied potentials ranged from 5 to 7.5 kV depending on the flow rate. The target pill was positioned under the emitter at a separation distance (z in Fig. 2) of approx. 55 mm. A stencil was placed on top of the pill to constrain deposition to a square region on the surface. The stencil was constructed from polyethylene terephthalate glycol (PETG) plastic (50 × 50 × 0.5 mm) with an 8 × 8 mm square opening in the center. During ePUF deposition, the pill was translated in a zig-zag pattern (illustrated in blue in Fig. 2) using motorized linear stages (not shown). The zig-zag pattern consisted of 8 passes with 1 mm pitch (spacing) between each pass (totaling 8 mm, the size of the opening in mask). Translation speeds ranged from 8 to 20 mm/sec. In this work, ePUF patterns were created on 200 drug tablets.

### Imaging

High quality reference images of the 200 ePUF patterns were taken using an 8-megapixel digital camera (ELP) equipped with a 5–50 mm varifocal lens and a LED ring light for illumination. Each reference image was captured orthogonal to the surface of the pills (20 mm from the pill surface to the lens) at a resolution of 2155 × 2155 pixels, and were labeled with the corresponding sample key to serve as the ground truth for pattern matching and authentication. The reference images (200 in total) were compiled and stored on a network drive to create the ePUF database for pattern matching. To mimic images taken at the point-of-care, a random selection of the ePUF patterns were captured using different cellphone cameras at various lighting conditions and different orientations.

### Pattern matching algorithm

**Figure 3:**
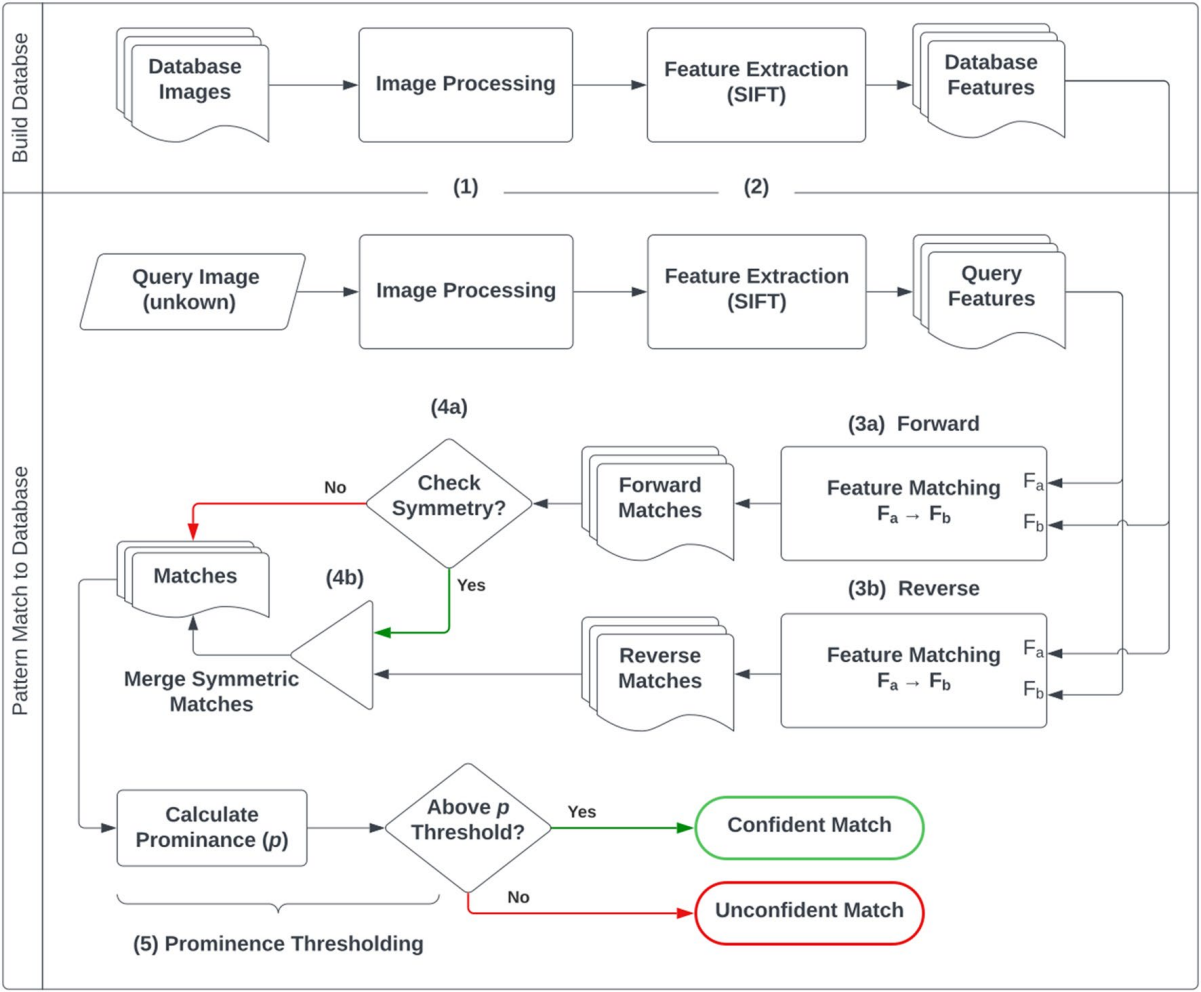
Process flow diagram for ePUF matching.

Machine vision was used for pattern matching and validation. Feature detection algorithms ORB (*Oriented FAST and Rotated BRIEF*)^35^ and SIFT (*Scale-Invariant Feature Transform*)^36^ were evaluated using the ePUF images stored in the database. While feature detecting with ORB was faster (due to the *FAST* keypoint algorithm and binary structuring), SIFT detected significantly more features within the images, resulting in superior matching accuracy. As such, SIFT was chosen to be the better candidate for this application. Figure 3 shows the process flow diagram of the pattern matching algorithm that was developed for this work. The *Build Database* operations (top row) are only required to be executed when a new ePUF is produced and needs to added to the database. The *Database Features* are computed once (per update to the database) and stored in memory to be quickly accessed for matching. The *Pattern Match to Database* section (bottom row) describes the process that is executed each time a new (unknown) pattern is to be tested against the database. The following paragraphs in this section provide details on each of the numbered steps presented in Fig. 3, and definitions of the algorithm nomenclature are presented in Table 1.

**Table 1:** Nomenclature for the pattern matching algorithm.

Variable	Meaning
$i, j, m, n$	Indices for incrementing
$a, b$	Denotes images $a$ and $b$
$F, F_{a} , F_{b}$	Features list (from images $a$ and $b$)
$f, f_{a} ,f_{b}$	Single feature (features list $F_{a}$ composed of multiple features $f_{a,i}$)
$D$	Feature descriptor (each feature $f$ has a descriptor $D$)
$d$	L2-Norm distance between two descriptors
$N$	Number of feature matches
$M\left( {f_{a} ,f_{b} } \right)$	Match from feature $f_{a}$ to feature $f_{b}$
$M^{\prime } \left( {f_{a}^{\prime } , f_{b}^{\prime } } \right)$	Reverse match from feature $f_{a}^{\prime }$ to feature $f_{b}^{\prime }$
$p$	Prominence threshold
$k$	Distance (Lowe’s) ratio
$\lambda$	Feature matching threshold

#### Image processing (pattern extraction)

**Figure 4:**
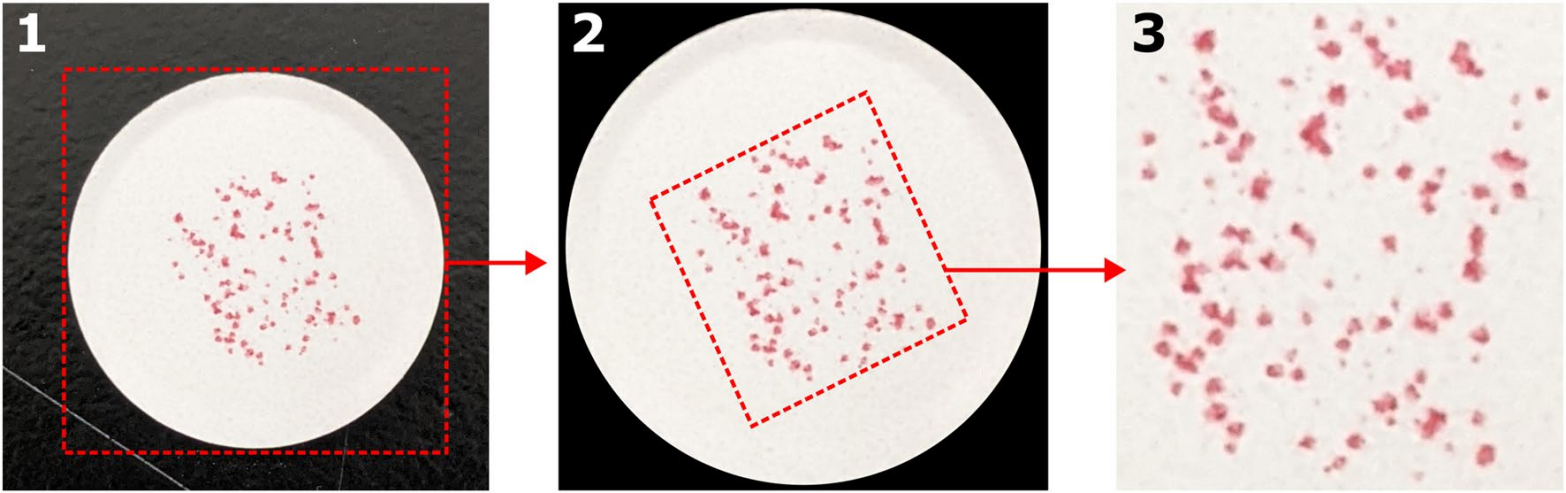
Image processing steps to extract ePUF pattern from an image of the pill. The raw image (1) is cropped to the pill and the background is removed (2), from which the ePUF pattern is extracted (3).

The images (both the unknown/query image and the known/reference image) are first processed in order to extract the ePUF pattern and optimize feature extraction (SIFT). The steps for image processing are briefly illustrated in Fig. 4. First, an auto-cropping algorithm is used to crop the raw image (Fig. 4, image 1) to the smallest bounding box that fits to the pill. Auto-cropping is performed by first converting the RGB image to HSV (hue, saturation, value) color-space and performing automatic Otsu thresholding on the value (brightness) channel. This results in a binary map with the pill region as ones and everywhere else as zero. The binary map is then used to fit a bounding rectangle and the image is cropped. Additionally, all the background pixels outside the binary map (within the cropped image) are set to black (shown in image 2). Next, Otsu thresholding is performed on the saturation channel (of the cropped image), resulting in a binary map where the dyed spots of the ePUF pattern are represented as ones. Finally, a bounding box is fit using the binary map of the pattern, and the image is rotated and cropped to the rectangle, resulting in a focused image of the ePUF (image 3). The cropped ePUF pattern (image 3) is converted to grayscale for SIFT feature extraction.

#### Feature extraction

After processing, the SIFT algorithm is used to detect key features within the images. Each extracted feature is represented by a keypoint, which records the central location of the feature, and a descriptor, which describes the texture in an area surrounding its respective keypoint. Each descriptor is represented as a vector of integers (128 in length). The number of features (keypoints and descriptors) that are extracted using SIFT depend on the image (i.e., dense vs. sparse texture) as well as various user-controllable parameters of SIFT (e.g., maximum allowed features).

#### Feature matching

Following SIFT, the computed features of the unknown query image and the features of the known database images are compared for matches. The L2-Norm (Euclidean distance) between feature descriptors is used to determine similarity (distance) between features in the query image and features in the database images. The L2-Norm is calculated using the following:

1 $$d = \parallel D_{1} - D_{2} \parallel _{L2} = \sqrt {\mathop \sum \limits_{n} \left( {D_{1, n} - D_{2, n} } \right)^{2} }$$

where $D_{1}$ and $D_{2}$ represent the two descriptors being compared, and $n$ denotes $n$-th component of each descriptor vector. For each feature $f_{a,i}$ in features-list $F_{a}$ the L2-Norm is calculated across all features $f_{b,j}$ from $F_{b}$. From the resulting list of descriptor distances (L2-Norms), the two lowest values are extracted, corresponding to the two features $f_{b,1}$ and $f_{b,2}$ that are most-similar to feature $f_{a,i}$. These two distance minima, $d_{1}$ and $d_{2}$, are then compared using Lowe’s ratio test ^37^:

2 $$\frac{{d_{1} }}{{d_{2} }} \le k$$

where $k$ is the distance ratio threshold. The ratio test is useful in eliminating ambiguous feature matches when features $f_{b,1}$ and $f_{b,2}$ have similar descriptor distances to feature $f_{a,n}$ (i.e., $d_{1} \sim d_{2}$). In these scenarios, the distance ratio will be close to unity, and they can be filtered out using an appropriate $k$-value (threshold). The remaining cases, where the distance ratio falls below the threshold, represent well-discriminated matches in which feature $f_{b,1}$ (which has the lowest descriptor distance $d_{1}$) is considered to be a good match to the feature $f_{a,i}$. This process is repeated for each feature $f_{a, i}$ resulting in a list of matches that link feature $f_{a, i}$ to a feature $f_{b,j}$ (i.e., match $M\left( {f_{a} , f_{b} } \right)$). For the forward feature matching step (3a), the features in $F_{a}$ are from the query image, and $F_{b}$ are from the database images. When symmetry checking (discussed further in the following section) is enabled, a separate reverse matching step (3b) is required. For this step, the features in $F_{a}$ are from the database images, and $F_{b}$ are from the query image. The effects of $k$ on matching accuracy is discussed below.

#### Symmetry checking

In some scenarios, the Lowe’s ratio test will not be sufficient to filter out ambiguous and incorrect matches, and additional filtering methods must be used. Here, we use a symmetry checking algorithm (also referred to as feature cross-checking) to deal with incorrect feature matches that are not handled using the Lowe’s ratio test alone. To perform symmetry checking, each forward match $M_{n} \left( {f_{a} , f_{b} } \right)$ from the set of forward matches is compared to each reverse match $M_{m}{\prime} \left( {f_{a}{\prime} , f_{b}{\prime} } \right)$ from the reverse matching step (3b). A match is considered to be symmetric (correct) when $f_{a} = f_{b}{\prime}$ and $f_{b} = f_{a}{\prime}$. If the symmetry criterion is satisfied, the match $M_{n} \left( {f_{a} , f_{b} } \right)$ is retained and the next ($n + 1$) forward match is tested. Feature matches that do not satisfy the symmetry criterion are eliminated.

#### Prominence thresholding

Feature matching (step 3) and symmetry checking (step 4; if symmetry checking is enabled) are completed for each feature set from the database (each database image has its own set of features), resulting in a separate set of matches between the query (unknown) image and each database image (i.e., 200 database images yield 200 sets of feature matches). Here, we define an additional metric we denote *prominence* to further constrain the matching algorithm and determine the confidence of pattern matching. Prominence thresholding is computed using the following two criteria:

3a $$N_{1} \ge \lambda *len\left( F \right)$$

3b $$\frac{{N_{1} - N_{2} }}{{N_{1} }} \ge p$$

Equation (3a) sets a threshold on the minimum number of feature matches that must be found between the query image and the database images, in which $N_{1}$ represents the maximum number of feature matches between the query image and an image in the database, and $\lambda$ is the thresholding parameter (from 0 to 1) multiplied by the maximum number of features ($len\left( F \right)$) in the query image (detected by SIFT). This criterion ensures that the number of feature matches between the query and database image is equivalent or greater than a percentage ($\lambda$) of the maximum number of detected features in the query image to rule out scenarios with very few features matches which are not a strong indicator of a good match. This work uses a matching threshold of $\lambda = 0.02$ to eliminate the cases where only a few feature matches are found. The feature-matching prominence is then evaluated with the criterion in Eq. (3b), where $N_{2}$ represents the second-largest number of feature matches between the query and database images ($N_{2} \le$ $N_{1}$), and $p$ is the prominence threshold. A $p$-value of unity represents the ideal scenario where there is only one image from the database that contains any feature matches to the query image, and thus the confidence of a correct image match (from the query image to an image in the database) is very high. A prominence threshold $p$ that is too low (i.e., close to zero) may lead to false positive matches, wherein the query image matches to multiple images in the database and the incorrect database image may be chosen as the best match. Conversely, a prominence threshold $p$ that is too high (close to unity) can be too restrictive and may lead to an unconfident match even when the query image matches to the correct database image. Much of the results presented in this work utilize a prominence threshold of $p = 0.5$. This was found to be the optimal value to eliminate false positives while not being overly restrictive.

Table 2 contains a list of possible outcomes from pattern matching. The rows highlighted in yellow (*confident*/*unconfident match*; also illustrated in Fig. 3) are the only two possible outcomes that the algorithm can produce when the ground truth match in the database is not known a priori. This would be the case in a point-of-care application wherein the algorithm would not have any information on whether or not an image captured by the patient exists in the database and thus cannot determine if its match is truly correct or incorrect. In these scenarios, the algorithm can only determine if a match found within the database is confident (above the prominence threshold) or unconfident (below the threshold). However, for our algorithm testing and evaluation (see sections “ePUF pattern authentication”, “Cross-validation of database images”, and “Feature matching specificity and optimization”) the ground truth match of each query image (Fig. 3) is known, which allows the algorithm to categorize the match as *correct* or *incorrect* (orange-highlighted rows of Table 2)*.* Unconfident matches are not tested to be correct or incorrect (i.e., they are simply categorized as unconfident), thus correct and incorrect matches both require a confident match to be categorized.

**Table 2:**
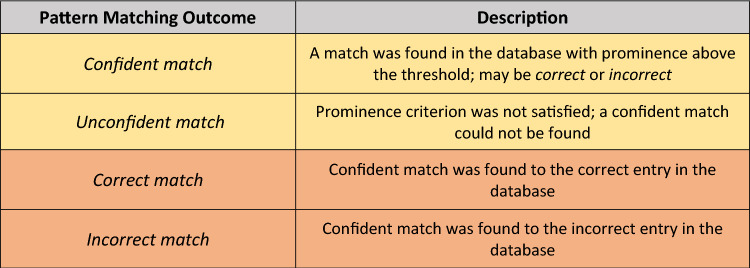
Descriptions of outcomes from the pattern matching algorithm.

Our pattern matching algorithm was implemented in Python using the *OpenCV* library for much of the heavy computational tasks (image processing, SIFT feature extraction, feature matching, etc.). The OpenCV algorithms are written in highly optimized C/C +  + and the overhead of calling them from Python is very minimal. Image processing and SIFT feature extraction took approx. 250 ms combined, per image. The computation cost of the feature matching and symmetry checking (steps 3 and 4 of Fig. 3) ranged from 1.5 to 4 ms per image depending on the number of SIFT features in the image and if symmetry checking is enabled (symmetry checking at least doubles the computation time). In total, the algorithm took approx. 1 s with symmetry checking (approx. 560 ms without symmetry checking) to check a single image against the database of 200 images. These computational times are for a desktop PC with a i7-6700 CPU (3.40 GHz) and 16 GB of RAM (2400 MT/s), without any GPU acceleration.

## Results and discussion

### Effects of electrospray parameters on ePUF structure

**Figure 5:**
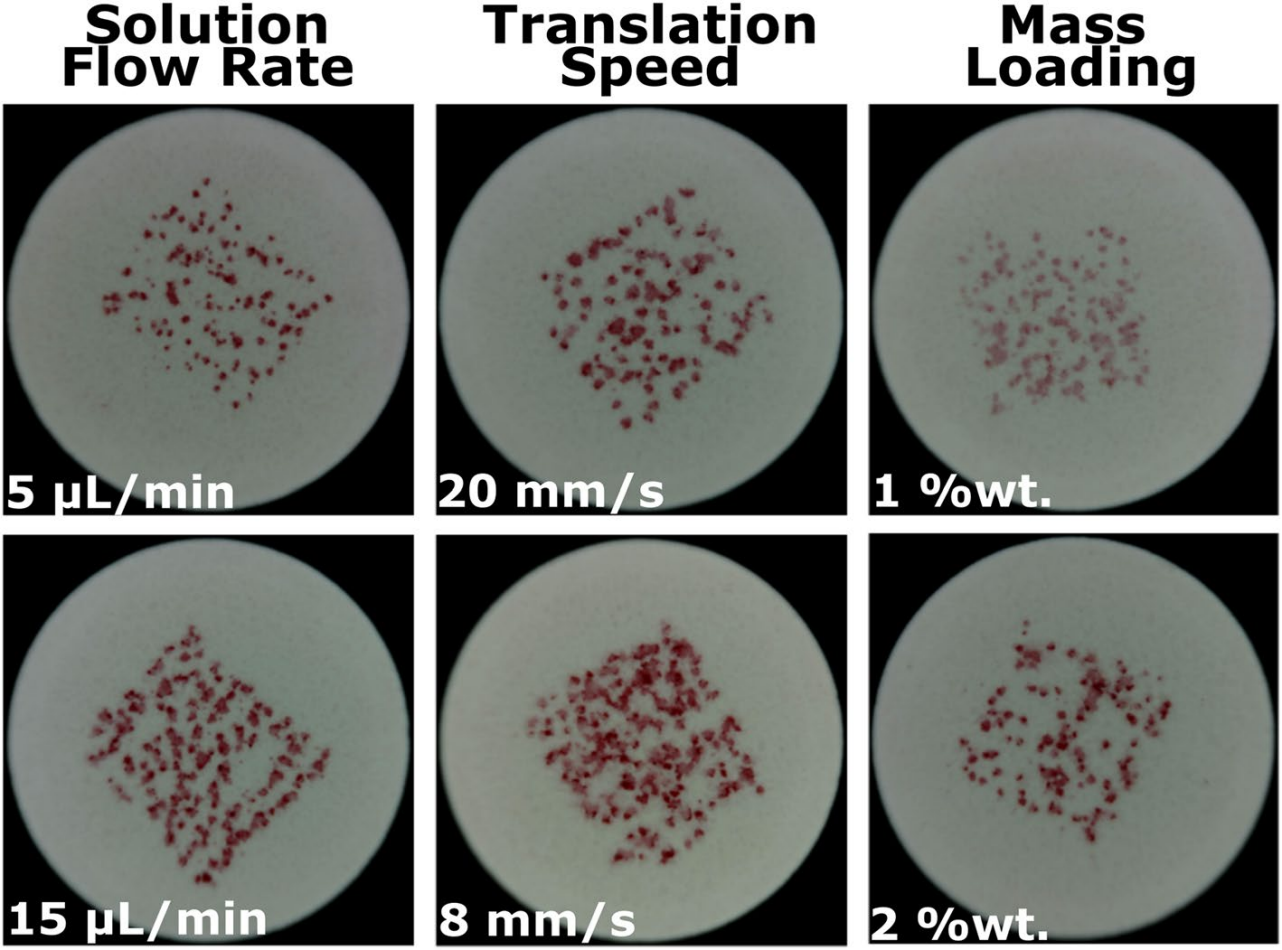
Effects of solution flow rate (left), translation speed (middle), and mass loading (right) on deposited ePUF pattern. Solution flow rate and translation speed affect the pattern density, and mass loading alters the color intensity of the pattern.

Electrospray deposition has many parameters that can be used to alter the deposition process and thus modulate the layout of the deposited pattern. Here, three primary control parameters are used to add variability to the ePUF patterns: (i) solution flow rate through the emitter, (ii) translation speed of the pill below the electrospray emitter, and (iii) mass loading of the dye. The effects of the three control parameters (solution flow rate, translation speed, mass loading) are visually represented in Fig. 5. The electrospray scaling laws from the works of Gañán-Calvo and De La Mora describe how the solution flow rate affects the size of the droplets emitted by electrospray^38–40^. As long as the physiochemical properties of the liquid remains constant (i.e., electrical conductivity, surface tension, permittivity) the droplets scale with solution flow rate, such that increases in flow rate produce larger initial droplets. This relationship (flow rate vs. droplet size) has an apparent effect on the structure of the patterns deposited onto the drug tablets, as can be seen in the left-most column of Fig. 5. Patterns deposited with a lower flow rate (e.g., 5 µL/min) produce a greater number of smaller droplets and sparsely-packed structure than those produced with higher flow rates (e.g., 15 µL/min). Additionally, the solution flow rate also plays a role in controlling the rate of droplet formation, such that higher flow rates produce a higher frequency of droplets. The density (sparsity) of the pattern is also heavily dependent on the translation speed of the target below the electrospray emitter, as shown in the middle column of Fig. 5. Using a fast translation speed (e.g., 20 mm/sec) produces droplet patterns that are more spaced apart. Slowing the translation speed (e.g., 8 mm/sec) causes the pattern to become more densely-packed as more droplets are delivered to the target. Though not shown here, flow rate and translation speed can be changed in-situ to further enhance the variability of the deposited ePUF patterns. The mass loading of dye in the ink affects the intensity of the deposited pattern (right-most column of Fig. 5). We note that mass loading is limited by the solubility of the dying agent in the carrier solvent; e.g., high values can lead to fouling of the emitter.

### ePUF pattern authentication

Authentication of the ePUF patterns was conducted using cellphone-captured images that were tested using the pattern matching algorithm against the database images. For these tests, 60 images of randomly chosen ePUF-labeled pills were imaged with different cellphones. All the cellphone images were captured near orthogonal to the tablet surface containing the ePUF pattern (non-orthogonal images are discussed in the next section). To be able to determine if the cellphone images matched correctly or incorrectly to the database, the sample number (i.e., ground truth) of each cellphone image was recorded and set aside. The images were then tested against the database using the pattern matching algorithm. For each pattern match (i.e., each cellphone image), the resulting sample number of the best match in the database (assuming the prominence threshold was met) was compared to the ground truth sample number that was recorded before pattern matching. Matches with identical sample numbers (i.e., ground truth/best match sample numbers are the same) are categorized as *correct*, and those with conflicting sample numbers are *incorrect*. In some scenarios, the prominence threshold criterion ($p$) was not satisfied. For these cases, they are deemed *unconfident* matches and a strong match in the database cannot be found.

**Figure 6:**
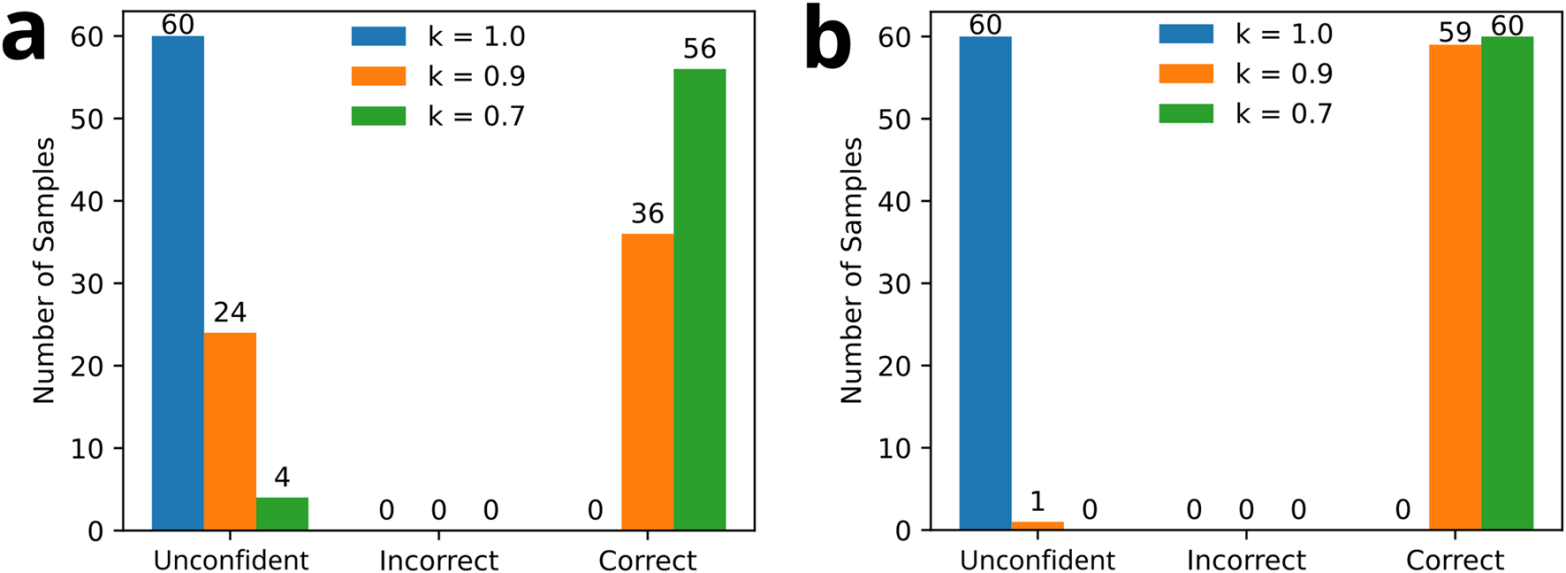
The effect of distance ratio $k$ on ePUF pattern matching. Plots (**a**) and (**b**) show the results of pattern matching without and with symmetry checking, respectively. Colored bars represent different values of $k$, as specified in the legends. A prominence threshold of $p = 0.5$ was used as the confidence criterion with a matching threshold $\lambda = 0.02$.

Plots showing ePUF pattern matching results for cellphone-captured images are presented in Fig. 6. The results in Fig. 6a were computed without feature symmetry checking, whereas the those in Fig. 6b included symmetry checking. The colored bars (blue, orange, green) show the effect on changing the distance ratio $k$ of the pattern matching algorithm. All pattern matching was computed using a prominence threshold of $p = 0.5$ and matching threshold of 2% ($\lambda = 0.02$). ePUF pattern matches with prominence values below the threshold are deemed as unconfident matches. Matches that satisfied the criterion were then checked to see if they were correctly matched (i.e., true positive) or incorrectly matched (i.e., false positive) to the database. For the results presented in Fig. 6 there were no patterns that incorrectly matched to the database.

Using a distance ratio $k = 1.0$ resulted in 0% confidence for pattern matching with and without feature symmetry checking enabled (Fig. 6a,b). Under this criterion, there were a significant number of ambiguous and/or incorrect feature matches that were not filtered out using the Lowe’s ratio test. As a result, the feature match prominence did not exceed the desired threshold and they are deemed unconfident. Using $k = 0.7$ transforms most (93%) of the unconfident matches into confident correct matches to the database. Enabling feature symmetry checking (Fig. 6b) produces even better results, with 98% correct matching at $k = 0.9$, and 100% at $k = 0.7$.

Pattern matching was also conducted between the database and itself (i.e., each of the 200 database images tested against all 200 images). Using symmetry checking, a distance ratio of $k = 0.7$, and prominence threshold of $p = 0.5$, we achieved 100% correct matches for the database images (i.e., each of the 200 database images only matched to itself).

**Figure 7:**
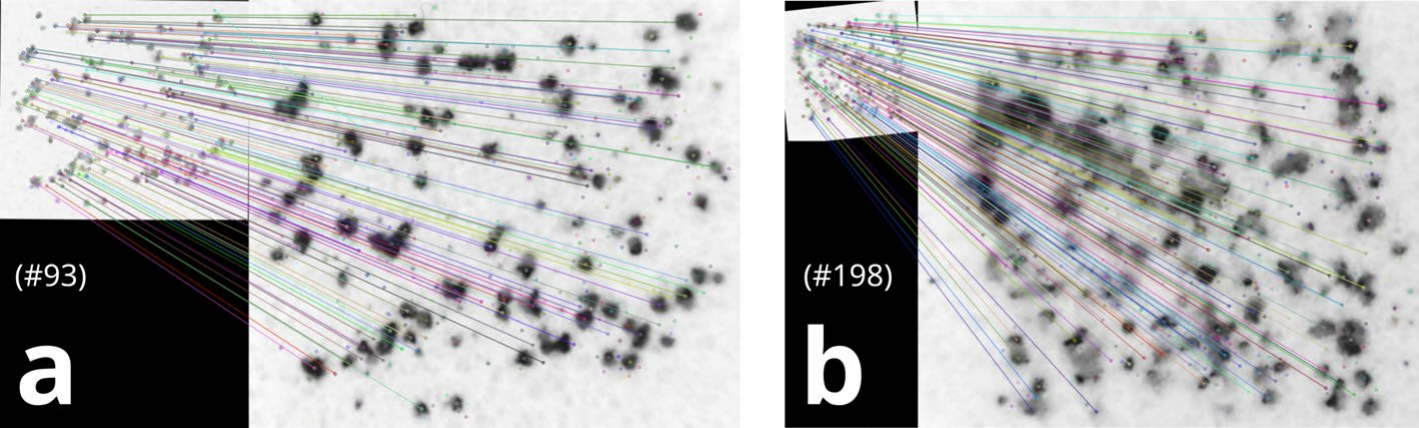
Representative images of correct ePUF matches between cellphone and database images. Top left (smaller) images show the ePUFs extracted from cellphone images. The larger images are the ePUFs from the database. The smaller (cellphone) image is rotated to match the orientation of the larger (database) image. The colored points show the SIFT features (keypoints) in each image, and the lines represent good feature matches between the images. Panel (**a**) shows the results for a less dense ePUF (pattern #93) and (**b**) shows the results for a dense ePUF (pattern #198). Pattern matching was conducted with distance ratio $k = 0.7$, prominence threshold $p = 0.5$, matching threshold $\lambda = 0.02$, and symmetry checking. Number of feature matches are 90 and 154 for (**a**) and (**b**), respectively.

Representative images of ePUF feature matches are shown in Fig. 7. The left panel (Fig. 7a) shows an example of feature matches for a sparse ePUF pattern and the right panel (Fig. 7b) contains an example of feature matches for a dense ePUF pattern. In each panel (a, b) the smaller image in the top left shows the image captured with the cellphone camera, and the larger image (on the right side of each panel) shows the matched image from the database. The colored lines represent individual matches between features of the cellphone image to features in the database image. Figure 7 clearly illustrates the robustness and accuracy of the SIFT feature extraction and pattern matching algorithm. The cellphone images were captured with different illumination and contrast, and at different resolution to the database images, however the algorithm could match them to their correct database image with a sufficiently high number of feature matches (90 and 154 for a and b in Fig. 7, respectively).

#### Pattern matching under non-ideal conditions

Testing under non-ideal conditions was conducted in order to evaluate the robustness of our pattern matching algorithm to changes in the illumination and orientation of the cellphone captured ePUF images. Eighteen images of various ePUF-labeled tablets were captured with a cellphone camera at different orientation, focal depth, and illumination. Similar to the procedure described in section “ePUF pattern authentication”, the sample label of each non-ideal cellphone image was recorded and the images were tested against the database with the pattern matching algorithm to determine if the correct matches could be found.

**Figure 8:**
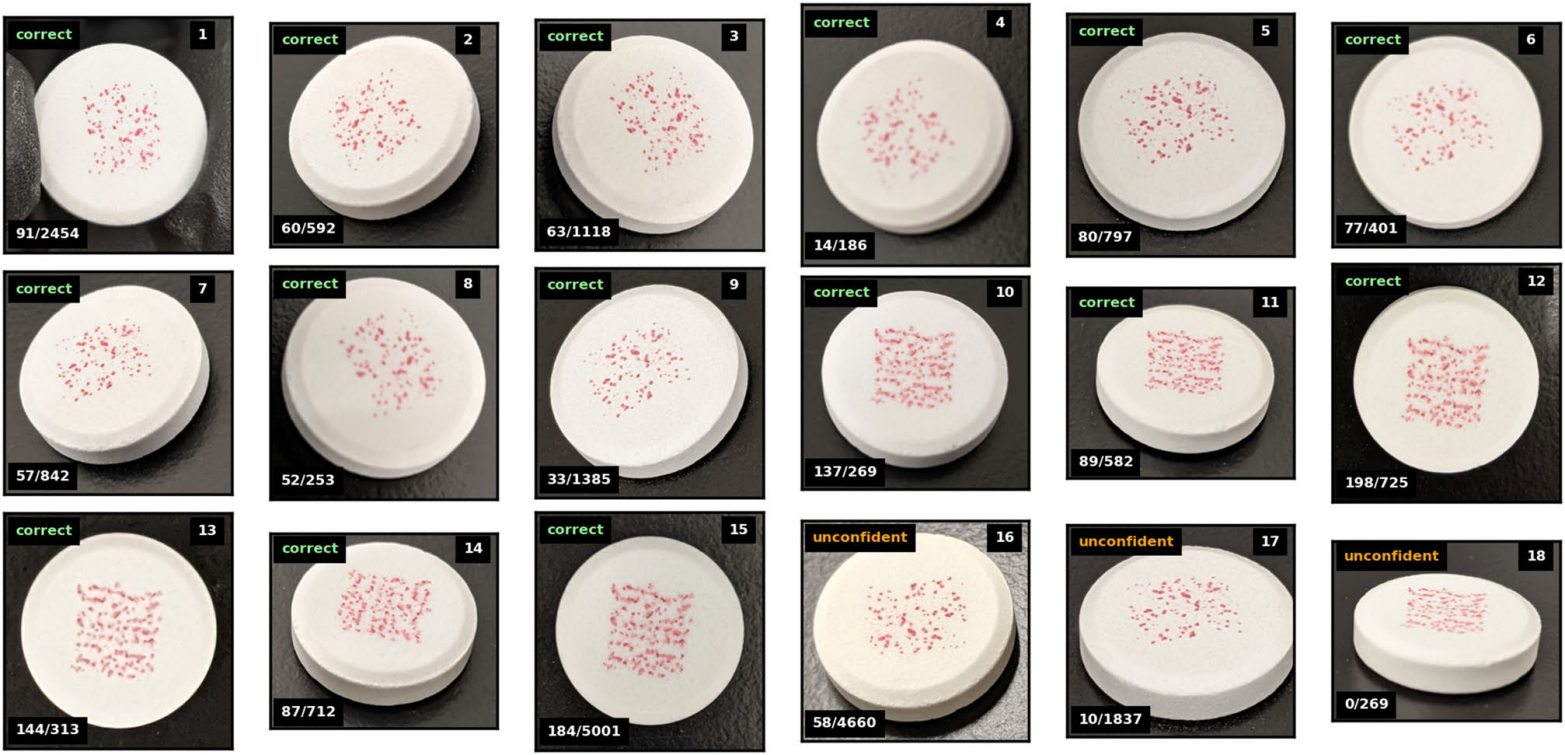
A collection of images captured with a cellphone camera at various orientations and lighting conditions to test the accuracy and robustness of the pattern matching algorithm. Pattern matching to the database images was conducted using $k = 0.7$ (distance ratio), $p = 0.5$ (prominence threshold), $\lambda = 0.02$ (matching threshold), with symmetry checking. Label in the top left (correct/unconfident) indicates if the image accurately matches with the database image for that tablet. Unconfident means the matching prominence fell below the threshold. Label in the bottom left is the number of correct feature matches (to the best match in the database), over the total number of features detected by SIFT for that image. Mean brightness of the images range from 130 to 200.

Figure 8 shows the collection of 18 stress-testing images of ePUF-labeled pills captured with a cellphone camera under various lighting conditions and orientations. The labels in the top left of each image indicate if the image was successfully matched with the correct corresponding image in the database. A label of *correct* corresponds to a 100% accurate match to the correct image in the database. The *unconfident* label means the algorithm was not able to find a match within the database with enough confidence (i.e., prominence/matching threshold criteria were not met). The labels in the bottom left indicate the number of individual feature matches between the features of each image to their corresponding best match in the database, and the total number of features detected for that image by the SIFT algorithm. From the images presented in Fig. 8, 15 out of 18 of them correctly matched to the database images with high confidence (i.e., high matching prominence). For most of these, the number of feature matches were over 50, and in a few cases reaching nearly 200. Figure 8 demonstrates the importance of camera angle on pattern matching accuracy and performance. Images that were captured close to orthogonal to the tablet had the greatest numbers of feature matches to their corresponding ground truth image in the database. As the angle becomes larger (images 17 and 18) the number of feature matches decreases, and when the angle is too large (exceeding 45° from the orthogonal axis; image 18) the ePUF pattern in the image becomes deformed and pattern matching is no longer accurate.

### Cross-validation of database images

**Figure 9:**
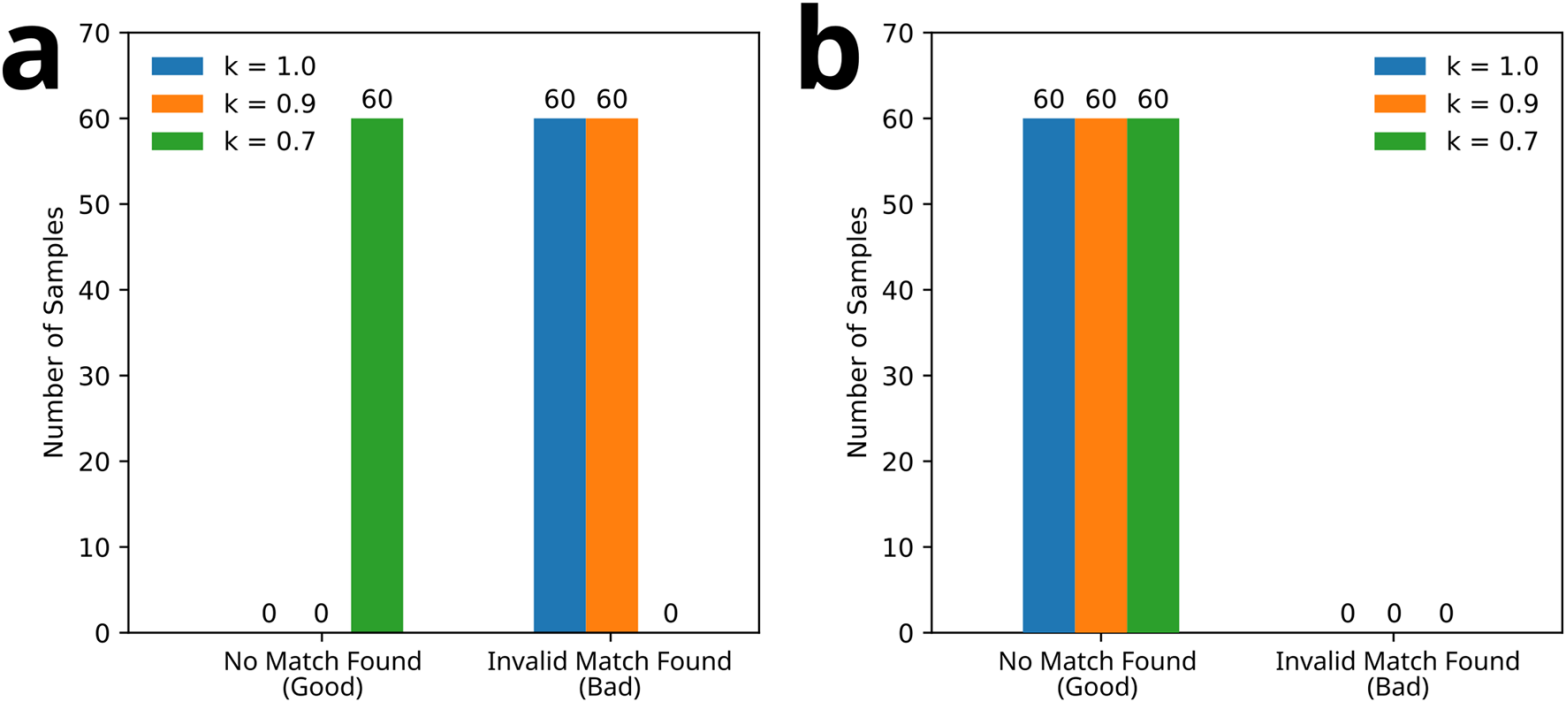
Cross-validation results for 50 randomly-chosen images from the database. Left plot (**a**) shows the pattern matching results with a prominence threshold $p = 0$ (i.e., no thresholding), and right plot (**b**) is with $p = 0.5$. All pattern matching (**a** and **b**) was done with symmetry checking enabled. Both plots were computed with a matching threshold $\lambda = 0.02$.

Cross-validation was conducted to test the pattern matching algorithm with ePUF patterns that weren’t registered in the database (i.e., false patterns). To do so, the corresponding true matches for the 60 near-orthogonal cellphone images (those used for the analysis in Fig. 6) were omitted from the database. Of the 60 cellphone images, 51 were captured from different samples (i.e., non-repeated images). Thus, after omitting the true matches from the database, 149 images remained in the database for pattern matching. The 60 cellphone images were then tested against the truncated database (149 images) to evaluate if the pattern matching algorithm could properly determine if they were unregistered (i.e., no match). The results of the cross-validation tests are presented in Fig. 9. The left plot (Fig. 9a) shows the pattern match results with no prominence threshold (i.e., $p = 0$), and the right plot (Fig. 9b) has a prominence threshold $p = 0.5$. The color of the bars represents different distance ratios used for during pattern matching, as labeled in the legends.

Figure 9 shows the significant effect of the prominence threshold when performing pattern matching with unregistered patterns. When there is no prominence thresholding (Fig. 9a) all 50 unregistered ePUF images incorrectly matched to images in the database. These images should not match any image in the database, and thus in this case they are deemed as invalid matches. In contrast, with a prominence threshold $p = 0.5$ (Fig. 9b), the pattern matching algorithm could not find a confident match (i.e., a match prominence above the threshold) within the database for any of the 60 images. Here, there is no effect of the distance ratio ($k$) on pattern matching for the unregistered images. The results in Fig. 9 were computed with symmetry checking enabled. However, tests were also run with symmetry checking disabled and the results were identical as those shown in Fig. 9. Thus, the prominence threshold ($p$) is the dominant parameter for these cases.

For the application of drug authentication, Fig. 9b shows the desired outcome for ePUFs not registered in the database (matches should not be found in the database). We note that the results in Fig. 9 are a specialized scenario, in which the unregistered patterns are known a priori to not exist in the database, so any matches to the database can be easily categorized as invalid. However, in the real scenario, there will be no knowledge of whether or not a pattern truly exists in the database, and it is up to the algorithm to decide. As such, any confident match (whether truly correct or incorrect) would pass the algorithm authentication, since the algorithm does not know if the pattern truly exists in the database. As such, using an insufficient prominence threshold (such as $p = 0$, as shown in Fig. 9a) with a false/unregistered pattern may result in a confident, but invalid match to a pattern in the database, which would incorrectly identify the false pattern as authentic. In contrast, using a higher prominence threshold (Fig. 9b) raises the confidence criterion of the algorithm, forcing the pattern match to be deemed unconfident and no match will be found if that threshold is not met. For the point-of-care application, an unconfident (no match) result is greatly favored over an invalid match, since an unconfident match can be flagged to the patient, whereas an incorrect match will indicate to the patient that the pill is erroneously authentic (i.e., false positive).

### Feature matching specificity and optimization

The specificity of individual feature matches (i.e., a feature in image A to a feature in image B) is crucial to ensure accurate ePUF authentication. If the feature-feature matching specificity is too low, there is a high probability of invalid or ambiguous matches which can result in a false negative or false positive ePUF match. As such, achieving high specificity is important to eliminate false feature matches and improve the accuracy of ePUF pattern matching. In our algorithm, the specificity of feature matches is dictated by the distance ratio $k$ and symmetry checking.

**Figure 10:**
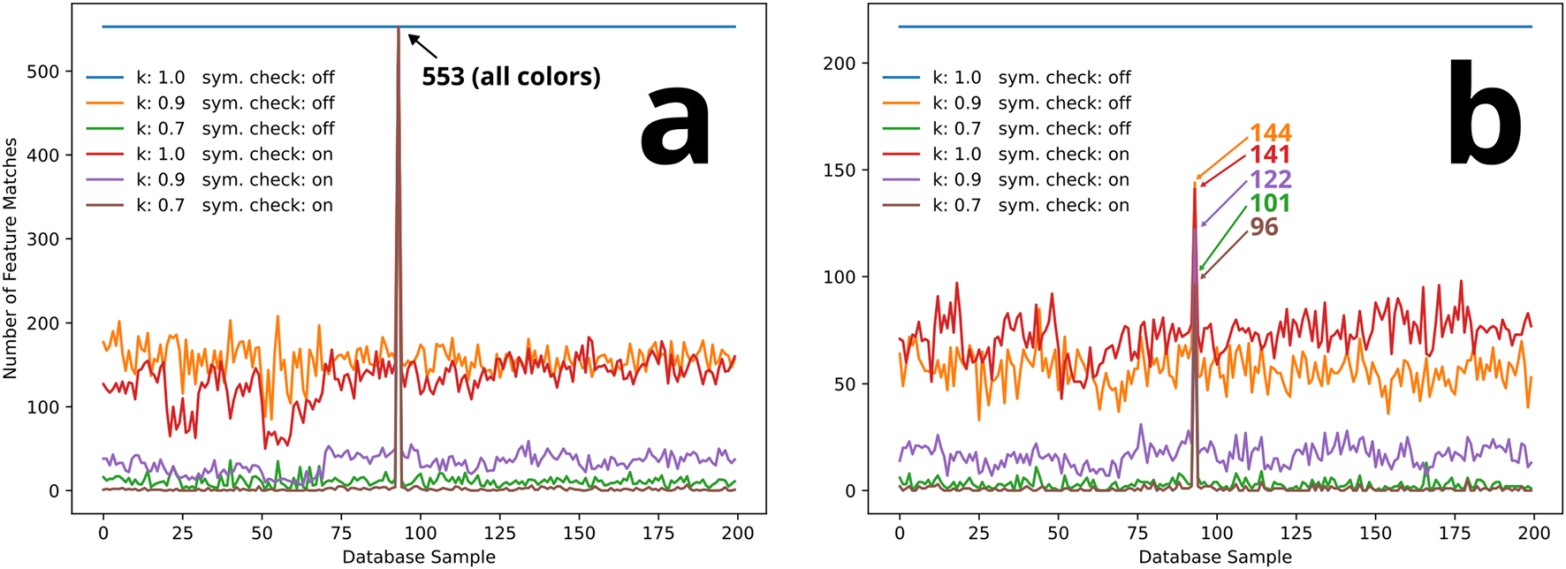
Effects of distance ratio $k$ and symmetry checking on specificity of feature matching. Plot (**a**) shows the effect for a sample image from the database, checked against the database images (200 in total). Plot (**b**) shows the effect for a cellphone image of the same sample (ePUF pattern #94; same ePUF pattern as in (**a**)) checked against the database images. Colored lines represent different feature matching conditions ($k$-value and symmetry checking) as specified in the legends. The peaks in (**a**) are all the same value (as labeled), and each of the peaks in (**b**) are labeled with their respective color.

Figure 10 plots the effects of distance ratio ($k$) and symmetry checking on feature matching. The y-axis is the number of individual feature matches between the query image and each of the 200 database images (shown on the x-axis). The colored lines (blue, red, green, etc.) show the results of different values of $k$ (distance ratio), with and without symmetry checking. Plot a shows the results for a database image and plot b shows the results for a cellphone image of the same ePUF pattern (#94).

In both plots, the blue line represents completely unconstrained matching. In this case, symmetry checking is disabled with a distance ratio $k = 1.0$, which causes a significant amount of ambiguous and incorrect feature matches. Unconstrained matching causes every feature of the ePUF pattern to match (either correctly or incorrectly) to a feature in each of the ePUF database images. In plots a and b this is illustrated as a horizontal line (blue) at a y-value that represents the maximum available features that SIFT detected in that image. Under these conditions, an ePUF match to the database cannot be confidently detected. Reducing the distance ratio to $k = 0.9$ (orange trace) filters out a significant portion of the ambiguous and incorrect feature matches, revealing a peak that represents the correct ePUF pattern match with the database. Enabling symmetry checking while leaving $k = 1.0$ (red trace) offers a similar trend. The most significant effect can be seen for $k = 0.7$ and symmetry checking enabled (brown trace), in which there is close to zero feature matches except for the peak. The plots in Fig. 10 also illustrate how the prominence $p$ is used to infer ePUF match confidence. For the case of unconstrained matching (blue trace), the number of feature matches is equal across all database images resulting in a prominence of zero. In comparison, for the well-constrained case ($k = 0.7$ and symmetry checking; brown trace), the peak prominence is close to unity (i.e., nearly zero feature matches across all 200 database images except for the peak) which infers a very strong and confident match.

**Figure 11:**
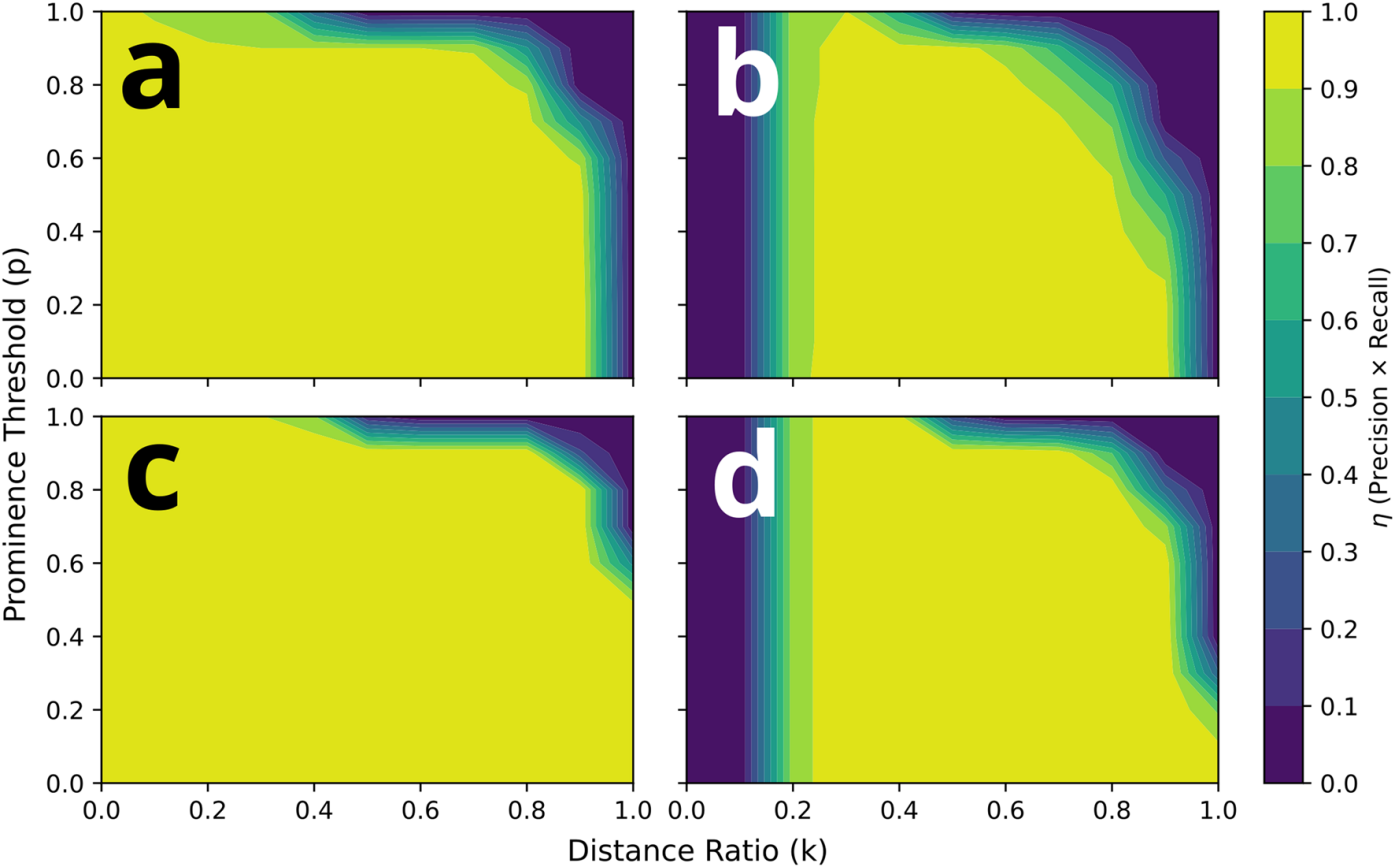
Heat maps showing the effect of distance ratio (in feature matching), and prominence threshold (for ePUF matching) for (**a**, **c**) the database images (checked against themselves), and (**b**, **d**) the cellphone images (checked against the database). Plots in the row (**a**, **b**) were computed without symmetry checking, and the bottom row (**c**, **d**) with symmetry checking. Colors represent different values of $\eta$ (product of precision and recall), as represented in the color bar on the right. Pattern matching was conducted without symmetry checking. All results computed with a matching threshold $\lambda = 0.02$.

The accuracy of ePUF pattern matching relies in part on the specificity of the feature-feature matches using the distance ratio and symmetry checking, but it can also depend on the match prominence $p$ and the prominence threshold that is used for pattern matching. Figure 11 are heat maps showing the effect of distance ratio and prominence threshold on ePUF matching accuracy. Plots a and b of Fig. 11 are for the database images (pattern matched against themselves) and the cellphone images (matched to the database), respectively. The colors in the heat maps represent different values of the $\eta$—parameter, which is a product of the *precision* and *recall* values. The precision is defined as $P = \frac{TP}{{TP + FP}}$ and recall as $R = \frac{TP}{{TP + FN}} ,$ where $TP$, $FP$, and $FN$ are true-positives, false-positives, and false-negatives, respectively. The values for precision and recall range from 0 to 1, thus the maximum value of $\eta$ is also 1. In the heat maps of Fig. 11, higher $\eta$—values indicate better pattern matching accuracy.

Figure 11 shows a noticeable difference in the effect of distance ratio and prominence for pattern matching of the cellphone images (plots b, d), compared to pattern matching of the database images (matched to themselves; plots a, c). Pattern matching for the database images is far less restrictive, in terms of the distance ratio and prominence threshold, than the cellphone images. This is to be expected, since the database images are being matched to themselves so only slight adjustment to the distance ratio is required to get completely accurate matches (i.e., $\eta = 1$). For the cellphone images, there is a smaller range of appropriate distance ratios from approx. 0.25 up to 0.9 without symmetry checking (Fig. 11b). A distance ratio of $k < 0.2$ is too restrictive (i.e., filters out too many feature matches) and reduces the accuracy, and $k > 0.9$ is not restrictive enough. Additionally, there is a greater dependence on the prominence threshold (y-axis). A prominence threshold that is too high (i.e., close to unity) is too restrictive for most distance ratio values. However, there is an optimal value at approx. $k = 0.3$ where high accuracy is achieved even at a very high prominence threshold (i.e., $p\sim 1$). With symmetry checking, the cellphone images (Fig. 11d) can be correctly matched to the database with $k = 1$ and $p = 0$ (i.e., no filtering). However, these constraints are not ideal for the point-of-care application in which falsified patterns will incorrectly (erroneously) match to the database, as shown in Fig. 9a. As such, we suggest a baseline prominence threshold of $p = 0.5$, for which a $k$-value from 0.3 to 0.8 is appropriate.

## Conclusion

In this work, we use electrospray deposition to deposit ePUF (edible-PUF) patterns onto drug tablets for identification and authentication against counterfeiting. The ePUF patterns were deposited onto drug tablets using an edible red dye, and images of the ePUFs were captured and stored in a database for authentication at the point-of-care. A machine vision pattern matching algorithm was developed to authenticate cellphone-captured images of the ePUF patterns to the database. We show how the algorithm parameters can be tuned and optimized to improve the specificity and accuracy of the pattern matching, and we were able to achieve very high accuracy in matching cellphone images of the ePUFs to their corresponding entry in the database (100% correct with good quality, near orthogonal images). Additionally, we show that the algorithm is robust to changes in illumination, orientation, and focus of the cellphone images. Cross-validation of the database images was used to test the algorithm against patterns that were not registered. Using the appropriate algorithm parameters, the unregistered patterns were correctly identified as unconfident matches to the database, which is the desired result for drug authentication.

The authors note that the throughput of our current process is modest, with 10–30 s required for ePUF deposition and approximately 60 s for database image acquisition. However, we envision that this technology can be scaled up using automation with multiplexed electrospray and imaging systems to achieve higher throughputs. While the target application of this technology was for anti-counterfeiting, we expect this same method could be employed for other pharmaceutical purposes including inventory control, expiration, and tracking.

## Data Availability

The datasets created and analyzed in this study are available in the Figshare repository, 10.6084/m9.figshare.25336396.
